# Biphasic Effect of *Phyllanthus emblica* L. Extract on NSAID-Induced Ulcer: An Antioxidative Trail Weaved with Immunomodulatory Effect

**DOI:** 10.1155/2011/146808

**Published:** 2010-11-07

**Authors:** Ananya Chatterjee, Subrata Chattopadhyay, Sandip K. Bandyopadhyay

**Affiliations:** ^1^Vijoygarh College Research and Development Centre, Kolkata 700032, India; ^2^Bioorganic Division, Bhabha Atomic Research Centre, Mumbai 400 085, India

## Abstract

Amla (*Phyllanthus emblica* L.), apart from its food value, can be used as a gastroprotective agent in non steroidal anti-inflammatory drug (NSAID)-induced gastropathy. It has been suggested that the antioxidative property of amla is the key to its therapeutic effect. Hence, on the basis of *in vitro* antioxidative potential, the ethanolic extract of amla (eAE) was selected for *in vivo* study in NSAID-induced ulcer. Intriguingly, eAE showed biphasic activity in ulcerated mice, with healing effect observed at 60 mg/kg and an adverse effect at 120 mg/kg.The dose-dependent study revealed that switching from anti-oxidant to pro-oxidant shift and immunomodulatory property could be the major cause for its biphasic effect, as evident from the total antioxidant status, thiol concentration, lipid peroxidation, protein carbonyl content followed by mucin content, PGE_2_ synthesis and cytokine status. Further, Buthionine sulfoxamine (BSO) pretreatment established the potential impact of antioxidative property in the healing action of eAE. However, eAE efficiently reduced pro-inflammatory cytokine (TNF-α and IL-1β) levels and appreciably upregulate anti-inflammatory cytokine (IL-10) concentration. In conclusion, gastric ulcer healing induced by eAE was driven in a dose-specific manner through the harmonization of the antioxidative property and modulation of anti-inflammatory cytokine level.

## 1. Introduction

*Phyllanthus emblica* L. or *Emblica officinalis *Gaertn, also known as Indian gooseberry, is a medium-sized deciduous tree of the Euphorbiaceae family. The fruits of *P.emblica* L. known as amla, are consumed as fruit, or in the form of food products. The species is native to India and also grows in tropical and subtropical regions. It primarily contains tannins, alkaloids, and phenolic compounds, but flavonoids derived from amla shows maximum beneficial in medicinal aspect. Ayurveda and Siddha systems of medicine have recognized the importance of this plant. It is one of the strongest rejuvenatives among Indian medicinal plants due to its antimicrobial [1, 2], antifungal [3], radioprotective [4], chondroprotective [5], antimutagenic, and anticancer properties [6, 7], but its most extraordinary features are its anti-inflammatory [3] and antioxidative properties [8]. A clinical study has also found that amla showed significant healing effect on gastric syndrome [9].

Among the total gastric ulcer patients, twenty-five percents suffer from acute drug-induced gastric ulceration [10, 11], a major example of which is gastric ulceration induced by non-steroidal anti-inflammatory drugs (NSAID) such as indomethacin. This kind of ulceration involves oxidative stress. The balance between proinflammatory and anti-inflammatory cytokines plays also an important role in various diseases. An imbalance in favour of the pro-inflammatory cytokines interleukin-1*β* or tumour necrosis factor-*α* has been implicated in the pathogenesis of different diseases including ulcerative colitis [12]. In addition, anti-inflammatory cytokines exert protective effects in experimental models of sepsis, rheumatoid arthritis, and autoimmune diabetes [13]. The present study highlighted that the balance of the pro- and anti-inflammatory cytokines could play also a significant role in NSAID-induced gastric mucosal injury. 

Treatment with indomethacin can substantially and drastically induce proinflammatory cytokine IL-1*β* and TNF-*α* levels, thus shifting the cytokine balance to the proinflammatory end, resulting in gastric ulceration. Amla extract (AE), however, has already proved itself a potent antioxidant, and different reports have claimed that its antioxidative property is significantly immunomodulatory [14]; hence, we examined the effect of AE on these parameters in the light of gastric ulcer healing.

## 2. Materials and Methods

### 2.1. Materials

Fruits of *Phyllanthus emblica* L. were collected from the local market and identified by the Botanical Survey of India (Ref. no. BSI /CNH/AD/Tech./2009).

### 2.2. Chemicals and Reagent

Indomethacin, thioberbituric acid (2-TBA), trifluro aceteic acid (TFA), omeprazole, 5-bromo-4-chloro-3-indolyl phosphate (BCIP), nitroblue tetrazolium (NBT), Tween-20, Bradford, buthionine sulfoxamine (BSO), 1,1-diphenyl-2-picrylhydrazyl (DPPH), 5,5′-dithiobis-2-nitrobenzioc acid (DTNB), hexadecyl trimethyl ammonium bromide (HTAB), and anti-dinitrophenyl (anti-DNP) antibody were purchased from Sigma-Aldrich Chemical Co, MO, St. Louis, USA. ethanol and methanol from E. Merck, Mumbai, India; bovine serum albumin (BSA), hematoxylin monohydrate, and eosin yellowish from Merck, Darmstadt, Germany; dimethylformamide (DMF) and tetramethyl benzidine (TMB) from Acros, Geel, Belgium; Prostaglandin E_2_ EIA kit and Total Antioxidant Status-Assay kit from Cayman Chemical; Cytokines TNF-*α*, IL-1*β*, and IL-10 ELISA kit from Pierce Biotechnology, Rockford, USA. Other reagents used were 35% hydrogen peroxide (H_2_O_2_) from Lancaster, Morecambe, UK; disodium hydrogen phosphate and sodium dihydrogen phosphate from BDH, Poole, Dorset, UK; Ecoline ALAT (GPT), Ecoline alkaline phosphatase, serum albumin, and bilirubin assay kits from Merck; serum glutamic oxaloacetic transaminase (aspartate aminotransferase) from Bayer AG, Wuppertal, Germany. All other chemicals used were of the highest purity available.

### 2.3. Animals

Male Swiss albino mice (6–8 weeks, 25 ± 2 g) bred in-house with free access to food and water were used for all of the experiments. The mice were kept in 12-h light/dark cycles and housed at 25°C ±  1°C. The animal experiments (*n* = 8) were conducted in accordance with the guidelines of the animal ethics committee of the Postgraduate Institute of Basic Medical Sciences, Kolkata, Animal Ethical Committee, Sanction no. IAEC/SB-3/2008/UCM-64 Dated-15/05/08-2011 and were handled following the International Animal Ethics Committee Guidelines, ensuring minimum animal suffering.

### 2.4. In Vitro Antioxidant Assay

DPPH (1-diphenyl-2-picrylhydrazyl) free radical scavenging assay.

An ethanolic solution of DPPH (100 *μ*M) was incubated with an ethanolic solution of each of the test samples at various concentrations, and the absorbance was monitored spectrophotometrically at 517 nm. The 50% reduction of DPPH absorbance at the given concentration of the test samples was used to calculate the IC_50 _ values [15]. The respective ethanol solutions of the test samples were used as the blank, while *α*-tocopherol was the positive control.

### 2.5. Drug Treatment

The ethanolic crude extract of amla and omeprazole [11] were prepared by using aqueous 2% gum acacia (gum acacia used as a vehicle) and were administered to the mice orally. In some cases, after ulcer induction, the mice were additionally treated intraperitoneally with BSO twice daily (first dose at a concentration of 5 mM was administered 4 h before drug treatment and the second dose of BSO at the same concentration was administered 3 h after the first dose).

### 2.6. Experimental Protocol for Ulceration and Assessment of Healing

Acute gastric ulceration in mice was induced by oral administration of indomethacin (18 mg/kg, single dose) dissolved in distilled water and suspended in 2% gum acacia as vehicle [16]. The animals were deprived of food but had free access to tap water 24 h, before ulcer induction. The normal and untreated control groups received the vehicle only throughout the course of the experiments. The treatment groups received different doses of amla crude extract (once daily) and omeprazole [3 mg/kg, once daily] [11] as positive control for different periods, with the first dose started 6 h after indomethacin administration. On the 1st, 2nd, 3rd, 4th, and 7th day the mice were sacrificed by cervical dislocation under anesthesia (ketamine, 12 mg/kg). The stomachs from the normal and treated groups were removed rapidly, opened along the greater curvature, and thoroughly rinsed with normal saline

### 2.7. Histological Analysis

The fundic stomach was sectioned for histological studies as well as damage score analysis. The tissue samples were fixed in 10% formalin and embedded in paraffin. The sections (5 *μ*m) were cut using microtome, stained with hematoxylin and eosin [17] and assessed under an Olympus microscope (BX41, Hamburg, Germany). The damage score (DS) was assessed [17] by grading the gastric injury on a 0–4 scale, based on the severity of hyperemia and hemorrhagic erosions: 0-almost normal mucosa, 0.5-hyperemia, 1-one or two lesions, 2-severe lesions, 3-very severe lesions, and 4-mucosa full of lesions (lesions-hemorrhagic erosions, hyperemia-vascular congestions). The sum of the total scores divided by the number of animals is expressed as the mean damage score. The experiments were performed by two investigators blinded to the groups and the treatment of animals.

### 2.8. Myeloperoxidase Assay

Myeloperoxidase (MPO) activity was determined following a reported method [18] with minor modifications. Whole gastric glandular portions of the stomach taken from all groups (100–150 mg) were homogenized in a 50 mM phosphate buffer (pH 6.0) containing 0.5% HTAB. This was followed by three cycles of freeze and thawing. The homogenate was centrifuged at 12000 × g for 20 min at 4°C. The supernatant (50 *μ*L) was collected for MPO assay and added to 80 mM phosphate buffer, pH 5.4, 30 mM TMB and 300 mM H_2_O_2_, to make a final reaction volume of 500 *μ*L. After the mixture was incubated at 25°C for 25 min, the reaction was terminated by adding 500 mM H_2_SO_4_ and the change in the absorbance was measured at 450 nm. Results were expressed as total number of neutrophils by comparing the optical density (OD) of tissue supernatant with the OD of mice peritoneal neutrophils processed in the same way.

A standard curve relating neutrophil numbers and absorbance was obtained by processing purified neutrophils and assaying the MPO activity with 0.0005% hydrogen peroxide as the substrate. The correlation between the number of neutrophils and units of MPO was determined using a reported technique [19]. One unit of MPO activity is defined as that converting 1 *μ*mol of hydrogen peroxide to water in 1 min at 22°C.

### 2.9. Assay of the Total Antioxidant Status in Serum

Total antioxidants were measured by the ABTS method using kits from CAYMAN as per manufacturer's instructions metmyoglobin (peroxidase) present in the chromogen provided in the kit reacts with H_2_O_2_ to form ferrylmyoglobin, a free radical species.The chromogen also contains ABTS (2,2′-amino-di-[3-ethylbenzthiazoline sulphonate]) which reacts with ferrylmyoglobin to produce a radical cation which has blue-green colour and can be measured at 600 nm. Antioxidants present in the added serum cause suppression of this colour production proportional to their concentration. Calibration of the assay was done using 6-hydroxy-2,5,8-tetra-methylchroman-2-carboxylic acid (trolox) equivalent.20 *μ*l sample and 1 ml of chromogen were required for the assay.

TAS was obtained using the formulae: TAS = factor × (absorbance of blank−absorbance of sample) mmol/l; factor = concentration of standard/(absorbance of blank-absorbance of standard).[20] 

### 2.10. Thiol Groups Assay

The thiol groups' (TSH) content was measured by the methods originally described by Ellman [21] and modified by Habeeb [22] with minor modifications. Whole gastric glandular portions of the stomach taken from all groups were homogenized in ice-cold phosphate buffer (pH 8.0). The tissue homogenate was then centrifuged for 10 min at 3000 rpm and the supernatant was collected for experiment. The supernatant (100 *μ*L) was added to a system containing 50 mM phosphate buffer, pH 7.4, protein dissolving solution and DTNB (0.1 mL, 10 mM) and was incubated for 15 min. The absorbance of the chromogen at 412 nm was read.

### 2.11. Assay of Lipid Peroxides

The extent of lipid peroxidation in tissue samples during *in vitro* incubation was measured by quantitating the amount of malondialdehyde (MDA) formed by 2-TBA reaction [23]. The amount of malondialdehyde produced was calculated using the molar extinction coefficient of MDA-TBA adduct as 1.56 × 10^5^ cm^2^mmol^−1^ [24].

### 2.12. Estimation of Protein Carbonyl 

The glandular stomach tissues from five animals were pooled, rinsed with appropriate buffer, and used for biochemical studies. The wet weight of the tissues was recorded, and experiments were carried out in triplicate. Glandular portions from the control, ulcerated and drug-treated mice taken at different time intervals were homogenized with a glass-Teflon homogenizing tube in 50 mM phosphate buffer (pH 7.4) and centrifuged at 1200 × g to obtain the supernatant.

The amount of protein carbonyls in the tissue homogenate was determined using the following method [25]. DNPH (4 mL,10 mM) in 2 M HCl was added to the supernatant (1.0 mL), which was incubated for 1 h with intermittent shaking. Ice-cold 20% aqueous TCA solution (5 mL) was added and the mixture incubated for 15 min. The precipited protein was washed three times with ethanol-ethyl acetate (1 : 1), then dissolved in 1 mL of a solution containing 6 M guanidine-HCl in 20 mM potassium phosphate (monobasic) adjusted to pH 2.3 with TFA. After centrifuging,the absorbance of the supernatant was read at 362 nm (*€* = 2.2 × 10^4 ^M^−1^cm^−1^)

### 2.13. Measurement of Protein Content

The protein content was determined by the Bradford method using BSA as the standard [26].

### 2.14. Mucin Assay

Following a reported method [27], the free mucin in the gastric tissues was estimated. Briefly, the glandular portion of the stomach was separated from the lumen of the stomach, weighed, and transferred immediately to 10 mL of 0.1% w/v Alcian Blue (AB) solution (in 160 mM sucrose solution buffered with 0.05 mM sodium acetate solution, pH adjusted to 5.8). After staining for 2 h, the excess dye was removed from the tissue by two successive rinses with 10 mL of 250 mM sucrose solution. The dye complexed with the gastric wall mucus was extracted with 10 mL of 500 mM magnesium chloride by intermittent shaking (1 min) at 30-min intervals for 2 h. The blue extract (2 mL) was vigorously shaken with an equal volume of diethyl ether. The resulting emulsion was centrifuged at 3600 rpm for 10 min, and the absorbance of the aqueous layer was read at 580 nm. The quantity of AB extracted per gram of wet glandular tissue was calculated from a standard curve prepared using various concentrations of AB.

### 2.15. PGE_2_ Assay

Following harvesting of the stomach, the corpus (full thickness) was excised, weighed (100 mg), and suspended in 10 mM sodium phosphate buffer, pH 7.4 (1 ml). The tissues were finely minced and incubated at 37°C for 20 min. After centrifugation (9000 × g), the PGE_2_ levels in the supernatant were measured by ELISA, following the Prostaglandin E_2_ EIA kit (Cayman Chemical) instructions.

### 2.16. Estimation of Tissue Cytokine Levels

The TNF-*α*, IL-1*β*, and IL-10 levels in the tissue homogenate were estimated using commercially available ELISA kits, following the manufacturer's protocol. 

The glandular part of the gastric mucosa after being washed with PBS containing protease inhibitors was minced and homogenized in a lysis buffer (10 mM Tris-HCl pH 8.0, 150 mM NaCl,1% Triton X-100,1 mL) containing leupeptin (2 *μ*g/mL) and PMSF (0.4 *μ*M). Following centrifugation at 15,000×g for 30 min at 4°C, the supernatant was collected and cytokines levels were measured. 

The samples along with the standards were seeded to each well at an appropriate dilution and incubated at room temperature for 90 min. The wells were washed (5 times), diluted polyclonal antibody (100 *μ*L) was added, and the mixture was incubated further for 2 h at room temperature. The wells were washed and incubated for 2 h after addition of HRPO conjugate (100 *μ*L) secondary antibody. After the final wash, TMB (100 *μ*L) was added to each well, the mixture was incubated for 15 min, the reaction was stopped by 1 N HCl, and the absorbance at 450 nm was read.

### 2.17. Toxicity Test

Toxicity tests were carried out for the subjected dose 60 mg/kg and a higher dose 300 mg/kg of amla in compared to sham-treated group for a period of 1 month. The levels of serum bilirubin, albumin, aspartate aminotransferase, alanine aminotransferase, blood urea nitrogen, and creatine kinase were measured using commercially available assay kits.

### 2.18. Statistical Analysis

Data are expressed as mean ± S.D. unless mentioned otherwise. Comparisons were made between different treatments (ANOVA) using the software GraphPad InStat (GraphPad Software Inc., San Diego, CA), where an error protecting multiple comparison procedure, namely Tukey-Kramer Multiple Comparison tests, was applied for the analysis of significance of all the data.

## 3. Results

### 3.1. Antioxidative Potential Encouraged to Select Amla Extract

IC_50_ values (*μ*g/mL) of test samples in scavenging DPPH radicals revealed that ethanolic amla extract (eAE) exerted 50% (*P* < .01) and 80.86% (*P* < .001) better antioxidative properties compared with methanolic (mAE) and hot water (hAE) amla extract, respectively (Table 1). Therefore, eAE was chosen for the evaluation of its antiulcerogenic property on NSAID-induced ulcerated mice. *α*-Tocopherol was used as positive control.

### 3.2. eAE on NSAID-Induced Ulcer

**Figure 1:**
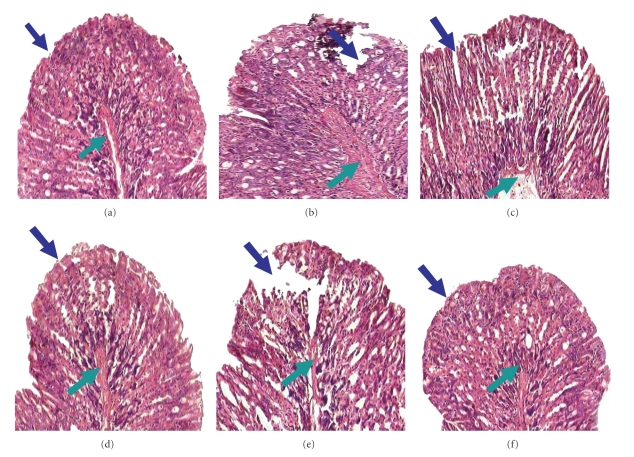
Histology of mouse gastric tissue after ulcer induction by indomethacin and the effect of eAE. Ulceration in mice was induced by indomethacin (18 mg /kg, p.o.). eAE at different doses (40 mg/kg, 60 mg /kg, and 120 mg/kg) and Omeprazole (3 mg/kg) were administered 6 h post ulcer induction as described in Section 2. At the third day of ulceration, mice were sacrificed, and the stomachs were sectioned for the histological studies. Histological photograph of sham-treated (a), ulcerated untreated (b), eAE-(40 mg /kg) treated (c), eAE-(60 mg /kg) treated (d), eAE-(120 mg /kg) treated (e), and omeprazole-(f) treated mice stomach section stained with hematoxylin and eosin, present here. Gastric tissue sections were photographed at a 40X magnification. Mucosal and submucosal layers are shown by blue and green arrows, respectively.

Antioxidants could be antiulcerous [28]. A study of ulcer index (UI) and MPO activity showed dose-dependent biphasic effect of eAE (Tables 2 and 3), which was further validated by histological study (Figure 1). A pick dose of 60 mg/kg eAE induced ulcer healing even at the third day of treatment, evidenced by reduced UI and MPO activity in a dose-dependent manner, thereafter with the increase of dose the healing effect gradually decline. The dose of 40 mg/kg b.w did not show healing effect possibly that dose is suboptimal to protect the stomach from injury but at the dose of (≥80 mg/kg), however, eAE aggravated the NSAID-induced ulcerated condition and delayed the healing process in a dose- and time-dependent manner compared with ulcerated control (Tables 2 and 3). These ulcerogenic biphasic properties of eAE were also validated by the study of total stomach mucin content (Figure 3). NSAID treatment significantly reduced mucin secretion at stomach compared with vehicle control, but eAE at the dose of 60 mg/kg induced mucin secretion on the third day of treatment. Intriguingly, at the dose of 40 mg/kg and 120 mg/kg eAE predominantly suppressed mucin secretion, significantly less compared with the sham treated. As eAE showed significant healing effect on the third day of treatment and biphasic ulcerogenic property was also evident on that very day, further experimental study was carried out on the third day only.

### 3.3. Oxidative Stress in Ulcer

**Figure 2:**
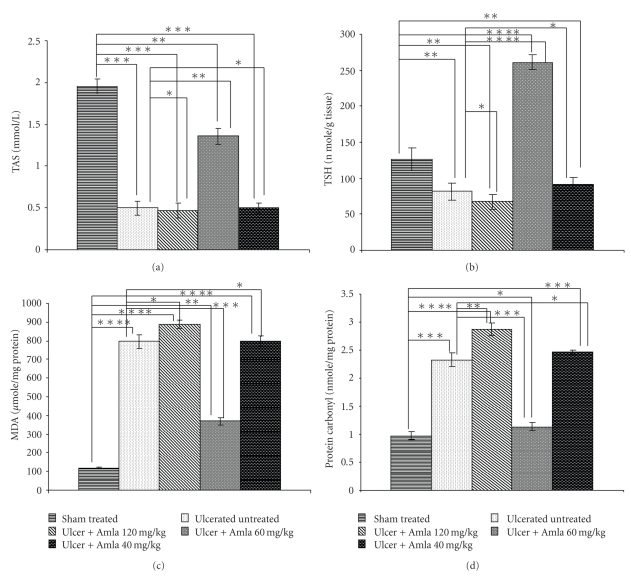
Effect of eAE at different doses (40 mg/kg, 60 mg/kg, and 120 mg/kg) on oxidative stress in NSAID-induced ulcer. Serum TAS (a), tissue total thiol (b), tissue MDA analysis (c), and tissue protein carbonyl (d) were carried out on 3rd day of ulceration, as described under Section 2. The values are mean S.D. ± (*n* = 8). *P > .05, **P < .05, ***P < .01, and ^  ∗∗∗∗^ *P* < .001.

**Figure 3:**
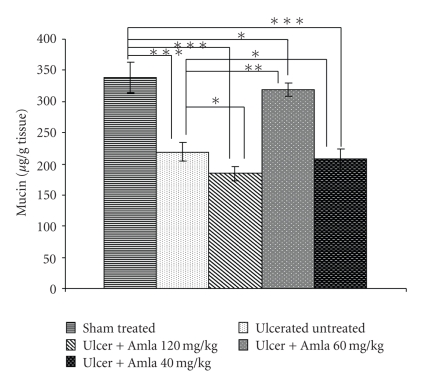
Effect of eAE at different doses (40 mg/kg, 60 mg/kg, and 120 mg/kg) on tissue mucin secretion in NSAID-induced ulcer. The mucin content of gastric tissues was carried out spectrophotometrically on 3rd day of ulcer, as described under Section 2. The values are mean S.D. ± (*n* = 8). *P > .05, **P < .05, ***P < .01, and ****P < .001.

Serum TAS (Figure 2(a)) and tissue total thiol (Figure 2(b)) content signified that oxidative stress was noteworthy in NSAID-induced gastric-ulcerated mice compared with vehicle control. On the third day of treatment, although eAE reduced oxidative stress at a dose of 60 mg/kg, it provoked oxidative stress at the dose of 40 mg/kg and 120 mg/kg, as further evidenced by the oxidative stress marker MDA (Figure 2(c)) and protein carbonyl (Figure 2(d)), which signified the oxidative injury of tissue lipid and protein.

### 3.4. eAE on PGE_2 _ Synthesis

**Figure 4:**
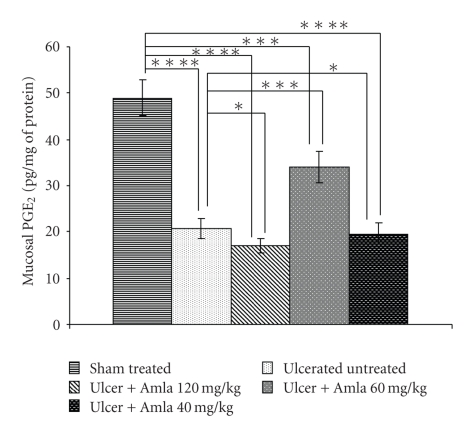
Effect of eAE at different doses (40 mg/kg,60 mg/kg and 120 mg/kg) on mucosal PGE_2_ synthesis in NSAID-induced ulcer. The mucosal PGE_2_ synthesis was carried out colorimetrically on 3rd day of ulcer, as described under ‘‘Materials and Methods”. The values are mean S.D. ± (*n* = 8). *P > .05, **P < .05, ***P < .01, and ****P < .001.

NSAID-induced ulceration suppressed PGE_2_ synthesis by 2.37-fold compared with vehicle-treated control. ELISA data revealed that at a dose of 60 mg/kg eAE induced PGE_2_ synthesis by 1.64-fold but at a dose of 40 mg/kg and 120 mg/kg, eAE reduced the PGE_2_ level 1.06 fold and 1.21-fold compared with ulcerated untreated mice (Figure 4), respectively.

### 3.5. eAE on Cytokines Expression

**Figure 5:**
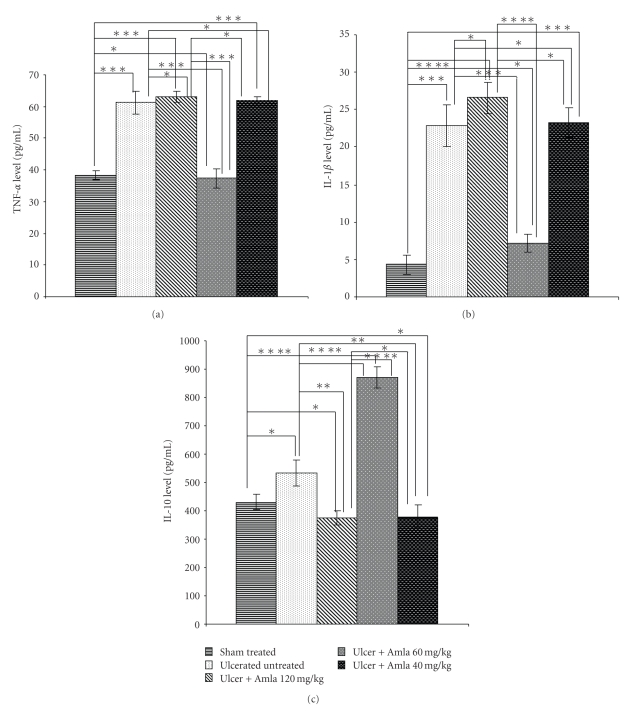
Effect of eAE at different doses (40 mg/kg,60 and 120 mg/kg) on tissue cytokines level in NSAID-induced ulcer. Tissue TNF-*α* (a), IL-1*β* (b) and IL-10 (c) level were analyzed colorimetrically by ELISA as described in ‘‘Materials and Methods”. The values are mean S.D.± (*n* = 8). *P > .05, **P < .05, ***P < .01, and  ****P < .001.

The balance of pro- and anti-inflammatory cytokines modulates NSAID-induced gastric ulceration. ELISA study showed that NSAID treatment induced TNF-*α* (Figure 5(a)) and IL-1*β* (Figure 5(b)) about 1.6- and 5.31-fold, respectively, compared with vehicle control. However, eAE at a dose of 60 mg/kg significantly reduced the level of inflammatory cytokines and induced the level of IL-10 (Figure 5(c)) by 1.63-fold compared with ulcerated untreated mice. However, treatment with eAE at a dose of 40 mg/kg and 120 mg/kg drastically induced inflammatory cytokine levels (TNF-*α* at 1.66 and 1.69- and IL-1*β* at 3.26 and 3.73-fold) and suppressed the anti-inflammatory cytokine level 2.29 and 2.32-fold compared with the 60 mg/kg dose of eAE respectively.

### 3.6. Antioxidant Property of eAE Modulated the Healing Action

**Figure 6:**
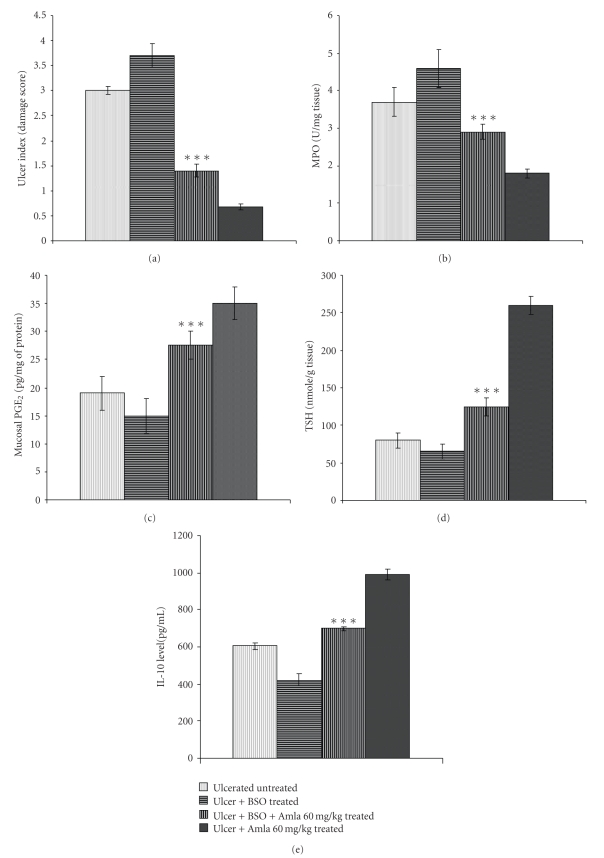
Antioxidant property of eAE modulated the healing action. The ulcerated mice were treated with eAE (60 mg/kg) alone or in conjunction with BSO (5 mM twice daily) for 3 days. The untreated and treated mice were sacrificed, and the ulcer healing was investigated from the DS (a), MPO activity (b), PGE_2_ status (c), total thiol content (d), and cytokine level IL-10 (e) as stated under Section 2. The values are mean S.D.± (*n* = 8). ***  *P* < .001 when compared with eAE-(60 mg/kg) treated group.

To evaluate whether the antioxidant property of eAE exerted a healing effect at a dose of 60 mg/kg, mice were pretreated with the glutathione S-transferase inhibitor BSO. UI (Figure 6(a)) and MPO activity (Figure 6(b)) were significantly increased after BSO pretreatment compared with the mice treated with 60 mg/kg eAE, followed by depletion of tissue PGE_2_ level (Figure 6(c)) and total thiol content (Figure 6(d)). However, BSO pretreatment did not have any impact on proinflammatory cytokine level (Supplementary Data) but drastically reduced the anti-inflammatory cytokine level (Figure 6(e)) compared with eAE, thus eliminating the healing effect of eAE.

### 3.7. Evaluation of Toxicity of eAE on Mice

Treatment with eAE at a dose of 60 mg/kg and 300 mg/kg to their respective group of mice for one month did not show any toxic side effect, as confirmed by comparing the values with sham-treated mice (Table 4).

## 4. Discussion

Antioxidants are known to augment the NSAID-induced gastric ulcer healing process as NSAID-induced ulcer formation involves oxidative stress [29]. On this light, previously we had been focused the cytoprotective nature of butanol extract of the water fraction of amla fruit on NSAID-induced ulcerated rats and reported that antioxidants are predominantly responsible for the cyto protective action [8]. In present paper, we have tried to established that amla possesses signinificant healing property against indomethacin induced stomach ulcers in mice at a biphasic manner on the basis of its antioxidative as well as immunomodulatory properties.

The *in vitro* study depicted ethanolic extract of amla as exerting the most antioxidative property compared with other solvent extracts, as validated by DPPH scavenging assay (Table 1). To evaluate the antiulcerogenic properties of eAE, NSAID-induced ulcerated mice were orally administered eAE once daily. Our microscopic examinations revealed that administration of indomethacin caused marked mucosal damage in the stomach within 6 h of ulcer induction. Maximum ulcerative damage was observed on the third day after administration of indomethacin (Table 2). Mice receiving only the vehicle showed no lesions in the gastric mucosa. Indomethacin (18 mg/kg) administration produced typical time-dependent acute mucosal lesions in mice, as assessed by histology (Figure 1). After seven days, however, the autohealing was evident.

MPO activity, a marker of neutrophil aggregation at the site of inflammation, is frequently increased under ulcerated conditions and reduced with the healing process [30]. Consistent with this, we also observed ulceration-induced MPO activity up to the third day, followed by its gradual reduction on the seventh day (Table 3).

At the same time, dose 120 mg/kg eAE aggravated ulceration (Tables 2 and 3) but at the dose of 40 mg/kg did not show any healing effect possibly that dose is suboptimal to protect the stomach from injury. The UI results were well supported by MPO activity data, where MPO activity was found to be appreciable at doses of eAE (40mg/kg and 120 mg/ kg) even on the seventh day compared with the ulcerated untreated mice. This established a strong contraindication of eAE use at its doses (40 mg/kg and 120 mg/kg) regarding gastric ulceration induced by indomethacin in mice. However, eAE at its standardized dose (60 mg/kg) showed a significant healing effect. The healing response was predominantly evident from day 3. On day 7, eAE treatment (60 mg/kg) showed a better rate of healing compared with ulcerated untreated mice (Table 3). 

Amongst various factors, oxidative stress has been implicated for the induction and pathogenesis of the indomethacin-mediated gastroduodenal injury [31, 32]. Extensive research has proved that antioxidants might be effective not only in protecting against gastric mucosal injury, but also inhibiting progression of gastric ulcer. This leads oxidative damages to lipids, proteins, and the thiol-dependent antioxidant defense systems [33, 34]. 

Experimental data clearly illustrated depletion of total thiol (Figure 2(b)) in ulcerated untreated mice, which was further validated by increased level of lipid peroxidation by MDA (Figure 2(c)) and protein carbonyl (Figure 2(d)). Nevertheless, the biphasic effect of eAE were also evident on manifestation of the oxidative stress markers. Treatment with eAE at a dose of 60 mg/kg predominantly reduced MDA and protein carbonyl level followed by a significant increase in total thiol, but exerted the opposite effect at the doses of 40 mg/ kg and 120 mg/kg.

Compared to the individual oxidative markers, TAS assay is based on the principle of inhibition of radical cation production by antioxidants in the sample. The concentration of antioxidant in the sample is inversely proportional to the absorbance of the radical cation produced by 2,2-azino-bis-(3-ethylbenzothiozoline-6-sulfonate) (ABTS) and ferrylmyoglobin. Measurement of total antioxidant activity in body fluids is of important prognostic and diagnostic value. The multiple defense system present in our body against damaging free radicals is collectively called antioxidants [35]. These antioxidants are present in the blood and are measurable. As measurement of individual component of the antioxidants is difficult and time consuming, it is easier to measure total antioxidants present in circulation.

Our results on the serum TAS level revealed severe oxidative stress in gastric tissue of the indomethacin-administered mice. Treatment with eAE at a dose of 60 mg/kg significantly increase serum TAS level, but exerted the reverse effect at the doses of (40 mg/kg and 120 mg/kg) (Figure 2(a)).

This biphasic nature of eAE also had an impact on immunological parameters. Stimulation of inflammatory cytokines is extremely important in mucosal defense. One of the most prominent modes of mediation of indomethacin-induced gastropathy is the increased expression of the pro-inflammatory cytokines [32, 36], as well as reducing the anti-inflammatory cytokines (IL-10) at the mucosal level. These led to a cytokine imbalance, which also correlate with the extent of ulceration. In ulcerated untreated mice, proinflammatory cytokine level increased significantly compared with the vehicle-treated group and simultaneously the anti-inflammatory cytokine level was repressed considerably (Figure 5). However, eAE treatment modulated the pro- and anti-inflammatory cytokine levels at a dose of 60 mg/kg. At this dose it reduced proinflammatory cytokines (TNF-*α* and IL-1*β*) and concurrently induced the level of tissue IL-10, thus exerting its healing effect. On the other hand, at a dose of 40 mg/kg as well as 120 mg/kg eAE reversed the cytokine balance to the proinflammatory end and thus once more proved the biphasic effect of eAE. 

As NSAID act as nonspecific cyclooxygenase (COX) blockers, treatment of NSAID-depleted tissue PGE_2_ level (Figure 4). ELISA data showed that eAE treatment induced tissue PGE_2_ level at a dose of 60 mg/kg, thus augmenting the healing process, but at a dose of 40 mg/ kg and 120 mg/kg PGE_2_ level was drastically reduced to 42.404% (*P* < .01) and 49.88% (*P* < .01) compared with the standardized dose (60 mg/kg) effect, respectively.

As eAE was screened on the basis of its antioxidative properties and treatment of eAE at a dose of 60 mg/kg markedly increased the total thiol level, we tried to delineate whether eAE-induced ulcer healing was modulated by its antioxidative property. To explore this issue, mice were intraperitonially injected with the glutathione S-transferase inhibitor BSO twice daily (see Section 2) before eAE treatment and further different healing parameters were measured. The role of endogenous sulphydryl compounds in mucosal protection has been demonstrated previously in ethanol induced gastric injury, in which the development of damage was accompanied by a decrease in mucosal sulphydryl compounds [37]. Sulphydryl compounds scavenge free radicals produced following tissue injury. BSO pretreatment predominantly hindered the healing effect of eAE (60 mg/kg) as substantiated by increased UI (Figure 6(a)) and MPO (Figure 6(b)) activity followed by significant suppression of PGE_2_ synthesis (Figure 6(c)). Further, its effect were also evaluated on cytokine levels where BSO pretreatment significantly repressed the IL-10 level while TNF-*α* and IL-1*β* concentration remained unchanged. This signified that although eAE treatment reduced inflammatory cytokine levels, the inhibitor study showed that tissue IL-10 level played a key immunomodulatory role for the augmentation of eAE-induced ulcer healing (Figure 6(e)). Concurrently, BSO pretreatment reduced the tissue thiol content (Figure 6(d)) compared with eAE-treated mice, signifying that eAE treatment drove augmented gastric ulcer healing through its antioxidative effect.

**Figure 7:**
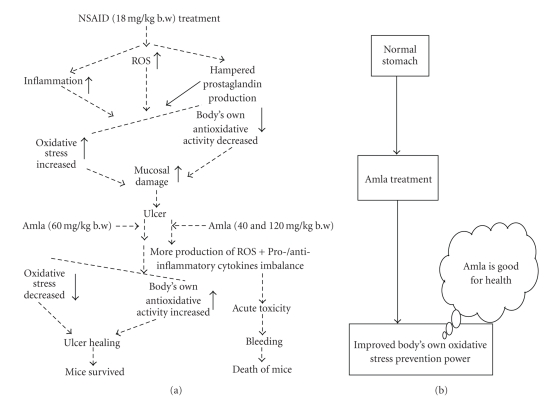
(a) Depicted that NSAID treatment provoked oxidative stress as well as inflammatory response and hampared prostaglandin production, resulting to formation of mucosal damage, ultimately ulcer. However, treatment of eAE at the dose of 60 mg/kg b.w helped to release the oxidative stress which in turn upregulate PGE_2_ status, IL-10 level.On the other hand treatment of amla at 40 mg/kg and 120 mg/kg b.w shows acute toxicity by provoking more ROS generation as well as imbalance pro/anti-inflammatory cytokines ratio. (b) Depicted that amla is good for health and it has no toxic effect in normal stomach at higher dose.

Therefore, it can be concluded that eAE can exert pronounced healing effect on NSAID-induced gastric ulcer on the basis of its antioxidant properties. Its healing effect, however, could be reversed to the ulcerogenic effect in a dose-specific manner, thus proving its biphasic action as well (Figure 7(a)). But amla, itself has no side effects even at its higher dose (Figure 7(b)). We evaluated the possible toxic effect of amla at the doses of 60 mg/kg and 300 mg/kg in mice (Table 4). The results suggested that amla given at this higher dose does not have any side effects in mice.

## Figures and Tables

**Table 1:** *In vitro *antioxidative assay.

Test sample	Concentration((*μ*g/mL)
Alpha tocopherol	25.98 ± 1.482
Ethanolic amla extract	3.0^∗∗∗, ##, ΨΨΨ^± 0.50
Methanolic amla extract	6.0***± 1.5
Hot water amla extract	15.67***± 1.528

IC_50_ values (*μ*g/mL) of test samples in scavenging of DPPH radical. Values are mean ± standard deviations (S.D.) (*n* = 3). ***P < .001 when compared to the standard alpha tocopherol, ^##^ *P* < .01 when compared to the methanolic amla extract, ^ΨΨΨ^ *P* < .001 when compared to the hot water amla extract.

**Table 2:** eAE on ulcer index.

Groups	Ulcer index (damage score) days of ulceration				
	1	2	3	4	7
Sham treated	0.00	0.00	0.00	0.00	0.00
Ulcerated untreated	1.667^∗, # ,h,j^ ± 0.577	2.33^∗, #,h,j^± 0.577	3.33^∗, # ,h,j^± 0.58	3.0^∗, #,h,j^± 1.0	1.3^∗, #,h,j^± 0.58
eAE (120 mg/kg)	2.0 ± 0.00	3.0 ± 1.00	4.0 ± 0.00	3.667 ± 0.58	2.667 ± 0.577
eAE (100 mg/kg)	1.667 ± 0.577	2.667 ± 0.574	3.67 ± 0.58	3.33 ± 0.577	2.33 ± 0.5774
eAE (80 mg/kg)	1.333 ± 0.577	2.0 ± 0.00	3.0 ± 1.0	2.67 ± 0.58	1.667 ± 0.58
eAE (70 mg/kg)	0.667^a^± 0.289	1.33^a^ ± 0.58	1.33^aaa^ ± 0.60	1.0^a^ ± 0.00	0.667^a^± 0.289
eAE (60 mg/kg)	0.667^a, ##^ ± 0.29	.816^aaa,####^±0.764	0.83^aaaa,####^±0.28	0.667^aa,###^±0.289	0.5^a, ###^± 0.00
eAE (50 mg/kg)	1.333^a^± 0.577	1.667^a^ ± 0.58	2.67^a^ ± 0.574	2.33^a^ ± 1.155	1.67^a^ ± 0.577
eAE (40 mg/kg)	1.7^a^ ± 0.58	2.33^a^ ± 0.58	3.3^a^ ± 0.60	3.3_a_ ± 0.68	2.4^a^ ± 0.577
Omeprazole(3 mg/kg)	0.833 ± 0.288	1.33 ± 0.60	2.33 ±0.58	2.0±1.0	1.667 ± 0.1638

The values are mean (*n* = 8) and (±) indicates S.D..*P < .001 when compared to the ShamTreated; ^a^ *P* > .05, ^aa^ *P* < .05, ^aaa^ *P* < .01, ^aaaa^ *P* < .001 when compared to ulcerated untreated; ^#^ *P* > .05, ^##^ *P* < .05, ^###^ *P* < .01, ^####^ *P* < .001 when compared to eAE (120 mg/kg); ^h^ *P* > .05 when compared to eAE (100 mg/kg);  ^j^ *P* > .05 when compared to eAE (80 mg/kg) treated group.

**Table 3:** eAE on MPO activity.

Groups	MPO activity (U/mg) days of ulceration				
	1	2	3	4	7
Sham treated	0.0153±0.003	0.0143 ± 0.005	0.0217 ± 0.003	0.021 ± 0.004	0.0224 ± 0.004
Ulcerated untreated	1.1454^∗,b,f,g^±0.074	1.8849^∗,b,f,ggg^ ± 0.061	2.7606^∗,bb,ff,ggg^ ± 0.047	2.6111^∗,bb,ff,gg^ ± 0.18	1.7655^∗,bb,ff,g^ ± 0.164e
eAE (120 mg/kg)	1.2417 ± 0.049	1.9407 ± 0.039	3.7863 ± 0.334	3.783 ± 0.313	3.6187 ± 0.042
eAE (100 mg/kg)	1.0868 ± 0.047	1.8523 ± 0.079	3.8357 ± 0.049	3.77 ± 0.119	2.9305 ± 0.036
eAE (80 mg/kg)	1.0825 ± 0.052	1.5424 ± 0.056	2.1369 ± 0.097	2.0766 ± 0.045	1.817 ± 0.045
eAE (70 mg/kg)	1.0079 ± 0.005	1.2408 ± 0.048	1.92 ± 0.031	1.853 ± 0.033	1.166 ± 0.047
eAE (60 mg/kg)	0.7585^aaa,bb^ ± 0.031	1.0599^aaa,bb^± 0.008	1.5363^aaa,bb^ ± 0.027	1.45^aaa,bb^ ± 0.053	0.8439^aaa,bb^ ± 0.038
eAE (50 mg/kg)	1.0223^aa^± 0.006	1.6236^aaa^± 0.017	2.3861^aa^ ± 0.009	2.3069^a^ ± 0.009	1.5302^aa^ ± 0.051
eAE (40 mg/kg)	1.0623^a^±0.038	1.5827^aaa^± 0.047	2.7629^a^ ± 0.015	2.7488^a^ ± 0.036	1.8709^a^ ± 0.081
Omeprazole(3 mg/kg)	1.0285 ± 0.008	1.3929 ± 0.009	1.9145 ± 0.029	1.755 ± 0.062	1.0359 ± 0.014

The values are mean (*n* = 8) and (±) indicates S.D. *P < .001 when compared to the Sham Treated;  ^a^ *P* > .05, ^aa^ *P* < .05, ^aaa^ *P* < .001 when compared to ulcerated untreated;  ^b^ *P* > .05, ^bb^ *P* < .001 whencompared to eAE (120 mg/kg);  ^f^ *P* > .05, ^ff^ *P* < .001 when compared to eAE (100 mg/kg);  ^g^ *P* > .05, ^gg^ *P* < .05, ^ggg^ *P* < .001 when compared to eAE (80 mg/kg) treated group.

**Table 4:** eAE on liver function tests in mice.

Groups							
	Parameters						
	Bilirubin mg/ml	Albumin g/dl	SGOT	SGPT IU/l	ALP	BUN mg/dl	CK IU/l
Sham treated	0.098 ± 0.016	3.19 ± 0.16	199.7 ± 2.421	86.82± 17.22	319.86 ± 8.16	16.06 ± 2.61	118.36 ± 20.0
Ethanolic amla extract (60 mg/kg)Treated	0.102 ± 0.012	3.25 ± 0.121	202.16 ± 2.984	85.01 ± 19.01	324.49 ± 5.94	18.72 ± 1.48	121.28 ± 17.69
Ethanolic Amla extract (300 mg/kg)Treated	0.099 ± 0.026	3.23 ± 0.182	202.93 ± 2.004	89.862 ± 17.36	324.85 ± 5.76	15.49 ± 1.92	123.25 ± 16.51

*n* = 25; ± indicates S.D.SGOT, serum glutamic oxaloacetic transaminase; SGPT, serum glutamic pyruvic transaminase; ALP, alkaline phosphatase; BUN, Blood Urea Nitrogen; CK, Creatine Kinase.

## Appendix

NSAID: Nonsteroidal anti-inflammatory drug,
ROS: Reactive oxygen species,
PG: Prostaglandin,
BSO: Buthionine sulfoxamine,
eAE: ethanolic amla extract,
TAS: Total antioxidant status,
ELISA: Enzyme-linked immunosorbent assay,
GSH: Reduced glutathione,
IL: Interleukin,
TNF: Tumor necrosis factor.
